# Inflammatory Complications of Intravitreal Anti-VEGF Injections

**DOI:** 10.3390/jcm10050981

**Published:** 2021-03-02

**Authors:** Jacob T. Cox, Dean Eliott, Lucia Sobrin

**Affiliations:** Retina Service, Department of Ophthalmology, Massachusetts Eye and Ear, Boston, MA 02114, USA; jacob_cox@meei.harvard.edu (J.T.C.); dean_eliott@meei.harvard.edu (D.E.)

**Keywords:** anti-vascular endothelial growth factor (anti-VEGF), intravitreal, intraocular inflammation, brolucizumab, bevacizumab, ranibizumab, aflibercept, post-injection, endophthalmitis, retinal vasculitis

## Abstract

Intravitreal injection of anti-vascular endothelial growth factor (anti-VEGF) agents is a commonly used therapy for numerous retinal diseases. The most commonly used of these medications are bevacizumab, ranibizumab, aflibercept, and brolucizumab. However, intravitreal administration of these agents is also associated with several inflammatory and non-inflammatory adverse events. The three inflammatory adverse events are sterile intraocular inflammation, brolucizumab-associated retinal vasculitis, and post-injection endophthalmitis. This narrative review summarizes the current literature regarding these conditions, including their epidemiology, presentation, management, outcomes, and pathogenesis. The inflammatory adverse events also share a number of overlapping features, which can make them difficult to discern from one another in a clinical context. This review discusses certain distinguishing features of these conditions that may aid providers in discerning between them and establishing the correct diagnosis.

## 1. Introduction

Intravitreal administration of anti-vascular endothelial growth factor (anti-VEGF) agents is the mainstay of treatment for multiple retinal diseases, including neovascular age-related macular degeneration (AMD), diabetic macular edema, and macular edema from retinal vein occlusions. There are several anti-VEGF agents on the market, including bevacizumab, ranibizumab, aflibercept, and brolucizumab. These medications are biologic drugs that inhibit the VEGF family of proteins, which are key to angiogenesis and vascular permeability. Each medication has ample high-quality evidence demonstrating its efficacy in treating select retinal vascular diseases, and they are the fastest growing therapy in ophthalmology [1].

However, inflammatory and non-inflammatory adverse events (AEs) have been associated with these medications. Non-inflammatory AEs include cataract formation, elevated intraocular pressure, retinal artery occlusion, ocular hemorrhage, and rhegmatogenous retinal detachment. The inflammatory complications of intravitreal anti-VEGF injection are sterile intraocular inflammation (SII), brolucizumab-associated retinal vasculitis (BARV), and infectious endophthalmitis.

SII and infectious endophthalmitis are both well-documented complications associated with all currently-available anti-VEGF agents. BARV is a newly-recognized AE that is specific to brolucizumab. The inflammatory AEs are difficult to discern from one another given their similar presentations. However, there are subtle differences that may aid in making the correct diagnosis. This is key given the potential for devastating outcomes if infectious endophthalmitis or BARV is not treated promptly, aggressively, and with an appropriate regimen.

The ubiquity of anti-VEGF therapy and its often prolonged course of repeated injections make it likely that retina specialists will face inflammatory complications. As such, one should be well versed in their presentation and management. The remainder of this review is dedicated to summarizing the inflammatory complications associated with anti-VEGF therapy, including presentation, treatment, outcomes, and means for establishing the diagnosis.

## 2. Anti-VEGF Agents

### 2.1. Bevacizumab

Bevacizumab (Avastin, Genentech, Inc., South San Francisco, CA, USA) is a full-length (Fab and Fc) humanized murine IgG1 monoclonal antibody against VEGF-1. It received U.S. Food and Drug Administration (FDA) approval initially in 2004 for treatment of colorectal cancer and subsequently for other malignancies, but it is used off-label to treat neovascular AMD and other retinal diseases. This is the most commonly utilized anti-VEGF agent per a 2018 ASRS survey, which found that bevacizumab was the first line-agent of choice in neovascular AMD for 70.2% of U.S. retina specialists [2]. This may be in part because it is the most cost-effective anti-VEGF medication [3].

### 2.2. Ranibizumab

Ranibizumab (Lucentis, Genentech, Inc., South San Francisco, CA, USA) is a recombinant, high-affinity humanized murine IgG1 monoclonal antibody fragment (Fab) targeting VEGF-A. It was approved in 2006 by the FDA for treatment of neovascular AMD. Its efficacy is similar to that of bevacizumab and aflibercept [4,5,6,7,8], although there is some low-quality evidence that it may reduce the central retinal thickness more effectively than bevacizumab in diabetic macular edema [9]. Its popularity as a first-line agent of choice in neovascular AMD amongst U.S. retina specialists is similar to that of aflibercept (12.8% vs. 16.4%, respectively) [2].

### 2.3. Aflibercept

Aflibercept (Eylea, Regeneron Pharmaceuticals, Inc., Tarrytown, NY, USA) is a recombinant fusion protein with VEGF receptors-1 and -2 fused to the Fc fragment of human IgG1. It was approved for use in neovascular AMD by the FDA in 2011. It has similar efficacy to bevacizumab and ranibizumab [8,10]. A randomized control trial suggested that aflibercept may have greater visual acuity benefit at one year in diabetic macular edema compared to bevacizumab or ranibizumab when visual acuity is poor at initiation of therapy [11].

### 2.4. Brolucizumab

Brolucizumab (Beovu, Novartis International AG, Basel, Switzerland) is the newest commercially-available anti-VEGF agent. It was approved by the FDA in October 2019 for use in neovascular AMD. Brolucizumab is a single-chain antibody fragment targeting all forms of VEGF-A. This medication was developed to reduce the treatment burden of anti-VEGF agents, as its high solubility and low molecular weight of 26 kDa allow for administration of increased molar equivalents compared to prior agents, which in turn may allow for longer intervals between doses. For instance, the HAWK and HARRIER Phase 3 trials found that 50% of patients could be maintained on 12-week dosing of brolucizumab with non-inferiority to eight-week dosing of aflibercept [12]. These trials also found that brolucizumab had greater efficacy in neovascular AMD as measured by resolution of subretinal and intraretinal fluid relative to aflibercept.

### 2.5. Pharmacokinetics

All of the anti-VEGF agents demonstrate “flip-flop” pharmacokinetics, in which the drug dissipates slowly from the globe but is then rapidly metabolized in the systemic circulation [13,14,15,16]. These medications are not actively metabolized intraocularly [17]. Christofordis et al. studied their intravitreal half-lives using positron emission tomography-computed tomography (PET-CT) to monitor intraocular concentrations of I^124^-labeled anti-VEGF molecules. The half-life was 4.2 days for bevacizumab, 2.8 days for ranibizumab, and 4.6 days for aflibercept [18,19]. Enzyme-linked immunosorbent assay (ELISA) analysis has demonstrated generally similar half-life values in non-vitrectomized eyes, with 4.3 to 9.8 days for bevacizumab [20,21,22], 2.3 to 7.2 days for ranibizumab [23,24], and 2.2 to 3.9 days for aflibercept [24,25]. Nimz et al. found the intravitreal half-life of brolucizumab to be 2.4 ± 0.3 days in non-human primates [26].

## 3. Sterile Intraocular Inflammation

### 3.1. Epidemiology and Presentation

SII (also termed pseudoendophthalmitis) is characterized by acute-onset intraocular inflammation without infection that resolves without antibiotic treatment. The reported incidence of anti-VEGF associated SII varies by study from 0.02 to 0.37% [27,28]. Presentation usually occurs between 24 h and seven days from the inciting injection [29,30,31]. Symptoms can consist of blurred vision, floaters, pain, and photophobia [31,32]. The most common of these are blurred vision and floaters [33]. Pain is only present in up to 46% of patients, and its presence is significantly associated with severe vitreous or anterior chamber (AC) inflammation [31,32,34]. However, most cases of pain are generally mild or moderate, with severe pain occurring less than 10% of the time [31,32,34]. Photophobia is also rare, occurring in four to 19% of patients [31,35].

Frequently, visual acuity is substantially diminished from its baseline at presentation, but it often returns to pre-injection levels after the inflammation resolves [29,31,33]. A retrospective study by Wickremasinghe et al. found a mean visual acuity of 20/87 at the time of injection, 20/272 at presentation, and 20/100 at the last follow up [29]. Similarly, data from Chong et al. demonstrated mean visual acuities of 20/114 at injection, 20/425 at presentation, and 20/143 at the last follow up [33]. Intraocular inflammation is present on exam in virtually every case. This may consist of vitritis, AC reaction, or most commonly, both [31]. Additional, less common exam findings include hypopyon, fibrin, keratic precipitates, corneal edema, conjunctival injection, and chemosis [31].

### 3.2. Treatment and Outcomes

Treatment is typically non-invasive, consisting of observation alone or topical corticosteroids. This can be supplemented with topical antibiotics, cycloplegics, or systemic corticosteroids. More invasive interventions have also been employed, including in-office vitreous tap, intravitreal antibiotics, and pars plana vitrectomy (PPV) [31,36]. SII is difficult to differentiate from infectious endophthalmitis due to their overlap in symptoms and exam findings, and many of these interventions are primarily used to rule out infectious endophthalmitis in cases where the diagnosis is uncertain. Hahn et al. found no difference in visual outcomes between non-invasive and invasive treatments for SII [34]. However, some evidence suggests that observation alone may be associated with higher rates of permanent vision loss compared to topical corticosteroid treatment [31]. Moreover, the retrospective nature of such studies may be confounded by selection bias given that invasive interventions such as PPV were likely reserved for severe cases.

SII resolves in two to 12 weeks on average [30,33,37], although this can take up to 15 weeks in some cases [31]. Visual acuity generally returns to baseline at seven to nine weeks [33] but can take longer, and visual acuity never returns to pre-SII levels in some patients. Greenberg et al. found that visual acuity declined from a mean of 20/50 at baseline to a mean of 20/178 at presentation in their cohort of aflibercept-related SII patients [31]. Yet visual acuity returned to a mean of 20/55 in all eyes where ocular inflammation had resolved by the study’s end, despite the fact that 15% of those eyes experienced a permanent two-line or greater decline in visual acuity [31]. Permanent visual acuity loss of two or more lines has been associated with rapid presentation, severely diminished visual acuity at presentation, the presence of fibrin, and older patient age [31,34].

### 3.3. Pathogenesis

Several models have been proposed for the mechanism underlying anti-VEGF associated SII. These generally revolve around an immune response to the medications themselves or to possible impurities in manufacturing, storage, or preparation [32].

Most patients have a history of prior treatment with the same medication, and the majority of patients who are reinjected with it do not develop recurrence. This suggests that most cases do not arise solely from patient-specific immunologic responses to the medication itself. Similarly, manufacturing issues are unlikely to account for the majority of cases because while some lots are associated with multiple SII cases, most cases are sporadic [34]. There are reports of single lot, same day administration to both eyes of a patient resulting in bilateral SII [31,34,38]. However, there is also at least one report of a patient receiving single lot, same day intravitreal anti-VEGF to both eyes who developed only unilateral disease [34]. This suggests that SII is a complex and multifactorial entity, since unifactorial issues such as manufacturing impurities, faults in provider preparation, or a patient-specific immune response would presumably have led to bilateral SII in that patient.

However, pharmaceutical production, distribution, or preparation is clearly implicated in some cases where select lots of anti-VEGF are linked to multiple SII cases. Manufacturer’s guidelines indicate that anti-VEGF agents should be maintained between two and eight degrees Centigrade, stored in their original carton until use, stored away from lit environments, and administered within eight hours of opening [29]. Deviation from this protocol could lead to degradation of the medication’s constituent compounds and prompt an immune response [39,40].

Wang et al. reported on 116 patients in Shanghai, China, who received injections from three counterfeit vials of bevacizumab. Of these, 80 patients (69%) developed post-injection SII. Vitreous samples from these patients demonstrated the presence of a bacterial endotoxin, which is presumed to be the causative agent behind these patients’ inflammation [36]. The bevacizumab subset of a retrospective study from Williams et al.’s demonstrated a bimodal distribution of SII, with the majority of cases occurring in 2008 and 2009 out of the eight-year study period [41]. Contaminants from the manufacturing process may be accountable for the high concentration of cases at that time, and six lots of bevacizumab were responsible for 36% (24/67) of all bevacizumab-associated cases [41]. In the same study, 69% (9/13) of aflibercept cases were associated with the same preparing technician, whereas neither of the other study medications (bevacizumab, ranibizumab) had more than 11% of cases associated with the same technician [41]. This may indicate that the preparation process for aflibercept injections is more susceptible to inducing SII than other medications, but with such a relatively small sample size, it is difficult to draw definitive conclusions. A biosimilar form of ranibizumab (Razumab) demonstrated a high rate of inflammatory complications when it was released in India in 2015 [42]. Ten percent of patients who received injections from the first three batches Razumab experienced post-injection inflammation [42]. An additional filtration step was then added to the manufacturing process, and endotoxin limits were corrected [43,44]. After these modifications, the rate of ocular inflammation was significantly reduced [45].

### 3.4. Differences between Bevacizumab, Ranibizumab, and Aflibercept

There is conflicting evidence regarding whether bevacizumab, ranibizumab, and aflibercept have significantly differing rates of SII. Some data suggest that aflibercept has a higher rate of SII than other agents. One Phase 4 study of 100 eyes treated with aflibercept or ranibizumab found that AC reaction of 0.5+ or greater (by the Standardization of Uveitis Nomenclature scale [46]) was seen significantly more often in eyes treated with aflibercept compared to ranibizumab (19% vs. 2%, respectively), although the incidence of vitritis did not differ significantly between the two groups [47]. However, a prospective study by Daien et al. featuring 11 SII cases from 88,150 total injection showed no statistically significant difference in SII rates between aflibercept and ranibizumab [27].

Retrospective and prospective data suggest that the rates of SII from bevacizumab are significantly higher than those of ranibizumab or aflibercept [27,41,48]. However, a meta-analysis by Sigford et al. found no statistically significant difference in the SII rates between these three agents when only analyzing prospective data [48]. Williams et al.’s retrospective study that found bevacizumab to have higher rates of SII compared to ranibizumab or aflibercept also interestingly noted that, of the 14 patients who developed SII with no history of a prior ipsilateral anti-VEGF injection, each one was a case of bevacizumab-associated SII; in contrast, all cases of ranibizumab or aflibercept-associated SII had received at least one prior ipsilateral injection of that same medication [41]. In this data set, the average number of prior injections with the agent in question was five, nine, and 13 for bevacizumab, ranibizumab, and aflibercept, respectively [41]. Thus, prior exposure may not play as key a role in the immunogenicity of bevacizumab as it does in ranibizumab and aflibercept. Alternatively, this could be explained by the fact that bevacizumab is often compounded at pharmacies while the other agents are distributed by the manufacturer in single-use vials. Any impurities introduced while compounding bevacizumab could lead to SII, even if it is a patient’s first exposure to the drug, whereas the distribution processes of ranibizumab and aflibercept may be less prone to such impurities. Thus, bevacizumab SII cases may represent a mix of cases resulting from impurities and cases resulting from true immune reactions to the bevacizumab molecule itself, while ranibizumab and aflibercept cases may consist more exclusively of immune responses to the drug molecules, which could be more dependent on prior sensitization.

If there truly is a higher rate of aflibercept- or bevacizumab-related SII compared to ranibizumab, then the reason behind this remains unclear. Instead of focusing on the bevacizumab compounding process discussed above, Souied et al. hypothesize it could be due to the Fc antibody portion on aflibercept and bevacizumab, which is lacking in ranibizumab [49]. However, this is speculative as SII is likely a multivariate and idiosyncratic reaction with differing etiologies in various cases, which would explain why it occurs both sporadically and in clusters and rarely recurs in patients who are reinjected with the medication. It is important to emphasize that not all publications bear out a statistically significant difference in SII rates between these medications.

### 3.5. Unique Features of Brolucizumab-Associated SII

Brolucizumab-associated SII is similar to that of other anti-VEGF medications, including similar symptoms, exam findings, treatment, and outcomes. However, some evidence suggests it has a more delayed course, with a mean of 24 days from injection to presentation [35]. The decrease in visual acuity at presentation (mean 20/67) is also less pronounced than that of SII from other medications. However, this may be confounded by the fact that many studies excluded patients who received antibiotic therapy and thus may have removed relatively severe cases [35]. As with other agents, final visual acuity was largely unchanged from baseline (mean 20/56) [35].

Brolucizumab has a higher rate of SII (>4%) than other anti-VEGF agents [50,51]. It is unclear why this medication results in higher rates of SII, especially given it does not contain an Fc fragment which is considered the possible immunogenic factor in aflibercept and bevacizumab. In brolucizumab’s case, the high rate of SII may result from its higher rates of anti-brolucizumab antibodies in serum compared to other anti-VEGF medications. The HAWK and HARRIER trials found these antibodies were present in 36 to 52% of patients even before initiation of brolucizumab therapy [12,52,53,54]. Once the treatment was started, this increased to 53 to 67% [12,52,53,54]. There was a higher rate of intraocular inflammation among those with these antibodies (6%) compared to those without (2%) [54]. By comparison, clinical trials of ranibizumab and aflibercept found antidrug antibodies in 0 to 3% of patients pre-treatment and in 1 to 9% of patients after a two-year treatment course [54,55,56]. However, the presence of these antibodies did not affect the drug’s efficacy and their clinical significance is unknown. The fact that several patients have received reinjection with brolucizumab after resolution of their SII without recurrence suggests this condition does not simply result from an adaptive immune response to the medication [35]. Interestingly, the Novartis Safety Review Committee found that 74% of intraocular inflammation cases occurred in the first six months of treatment, while none of the cases occurred in the final six months of the two-year trial [12,51].

## 4. Brolucizumab-Associated Retinal Vasculitis

BARV is an inflammatory AE that is specific to brolucizumab [57]. Given that brolucizumab only received FDA approval in October 2019, it is unsurprising that this newly recognized entity remains poorly understood. It is unclear if BARV represents a severe presentation on the same spectrum as SII or if it is a unique entity altogether.

### 4.1. Post-Market Surveillance and Reporting

In February 2020, only months after brolucizumab’s FDA approval, the American Society of Retinal Specialists (ASRS) announced it had “received reports of [brolucizumab-associated] inflammation which included more than a dozen cases of vasculitis, of which greater than two-thirds were designated as occlusive retinal vasculitis by the reporting providers” [58]. Early case reports outlined post-injection inflammation and retinal vasculitis that was often occlusive and associated with significant vision loss [59,60].

These reports prompted Novartis Pharma AG to assemble an independent safety committee comprised of nine members, two separate external data monitoring committees, and an independent observer from ASRS to conduct a post hoc analysis of the HARRIER and HAWK trials [61]. Their analysis found that of the 1088 eyes treated with brolucizumab from 1088 patients, 36 developed “probable or definite” retinal vasculitis (3.3%). Each case featured concomitant intraocular inflammation and the retinal vasculitis was occlusive in 24 of the 36 cases (67%). At 3.3% and 2.1%, respectively, the rates of brolucizumab-associated retinal vasculitis and vascular occlusion were significantly higher than those reported in the original HARRIER and HAWK studies. However, despite the potential for vision loss from these events, the overall rate of moderate-to-severe vision loss (≥15 ETDRS letters) in the HARRIER and HAWK trials was similar between brolucizumab (7.4%) and aflibercept (7.7%) [62].

### 4.2. Epidemiology and Presentation

Despite the Novartis safety committee’s report, the exact incidence of BARV remains unknown. The expanded 96-week safety outcomes from the HAWK and HARRIER trials found that six of the 730 brolucizumab patients (0.8%) experienced intraocular inflammation-associated retinal artery occlusion or thrombosis [63]. A review of ASRS data indicated that 14 cases (11 of which were occlusive) had been voluntarily reported by providers out of the 46,000 brolucizumab injections that were administered in the United States to that date, suggesting an incidence of 3.0 cases per 10,000 injections [58] Similarly, post-market data from Novartis suggested the incidence of occlusive retinal vasculitis was 4.7 per 10,000 brolucizumab injections [64]. Importantly, these data may underestimate the true incidence compared to the Novartis safety committee’s report given their reliance on voluntary reporting.

Reviews thus far suggest that brolucizumab-associated retinal vasculitis has a strong female predominance (88–100%) [54,57]. Age and ethnicity are generally consistent with the neovascular AMD population at large, with a mean age of 77 to 79 years and 92 to 96% Caucasian ethnicity [54,57]. Underlying cardiovascular disease (e.g., hypertension, diabetes mellitus, cardiac arrhythmia) may be a risk factor for BARV. All patients who experience occlusive vasculitis in the HARRIER and HAWK trials had underlying cardiovascular disease, and the same is true for the majority of patients in another retrospective case series [57,63]. Otherwise, no pattern of medical history, ocular history, or medication allergy has been identified [54,57,59,60]. Rarely, patients are noted to have a history of cancer or autoimmune disease (e.g., multiple sclerosis, Graves’ disease, or Raynaud syndrome), but these comprise only a minority of BARV patients [54,57].

Symptom onset can range from seven to 56 days after the most recent brolucizumab injection [54,57,59,60]. The hallmark symptoms are blurry vision (58–62%), floaters (46–67%), redness (19%), pain (17–31%), and scotoma (12–25%) [54,57]. Visual acuity is usually decreased at presentation, from an average of 20/53 at the antecedent brolucizumab injection to a mean of 20/191 at diagnosis [57]. Intraocular inflammation is usually present on exam (92–100% of patients); inflammation can be localized to the AC (0–31%), vitreous (27%), or both (35–73%) [54,57]. Fine keratic precipitates, conjunctival injection, and Descemet folds are also seen in a minority of patients, but hypopyon has not been reported in this condition.

Clinical evidence of vasculitis is usually, but not always, present at presentation. A minority of patients may present with isolated intraocular inflammation only to return with new findings of vasculitis at follow-up, even if corticosteroids were started and the intraocular inflammation has decreased [57]. Vasculitis can involve arteries, veins, and capillaries. Large- and small-caliber retinal arteries can be affected, demonstrating any combination of narrowing, occlusion, and perivascular sheathing. Signs of retinal ischemia include retinal whitening, cotton wool spots, intraretinal hemorrhage, and pericentral acute middle maculopathy. Optic nerve swelling, phlebitis, and venous dilation or narrowing can also be seen on fundus exam [57]. Occlusive features of vasculitis are seen in 67–85% of cases [54,57].

### 4.3. Diagnosis, Treatment, and Outcomes

The diagnosis can be established in patients who have received intravitreal brolucizumab within the past 8 weeks, where clinical evidence of vasculitis is present, and if infectious endophthalmitis is unlikely. In cases where only signs of SII are present, close follow-up is recommended, and serial fluorescein angiography (FA) with peripheral sweeps should be considered to assess for evidence of subtle vasculitis or retinal occlusive events [62]. However, the therapeutic effect of brolucizumab may diminish some vasculitis-associated FA findings, such as hyperfluorescence and staining of the vessel wall or fluorescein leakage. As such, these findings may become more appreciable on serial imaging as the medication is metabolized. Alternatively, BARV is specific to brolucizumab and should not be considered in patients who have not received that medication. Hypopyon, severe pain, or severe injection are suggestive of infectious endophthalmitis. In these patients, it may be appropriate to default towards an infectious treatment regimen even if vasculitis is present.

Most BARV patients are treated with topic corticosteroids, systemic corticosteroids, or a combination of the two. Some patients may require additional sub-Tenon’s or intravitreal corticosteroid injections. Rarely, patients receive pars plana vitrectomy (PPV) as part of their management. While PPV offers the theoretical benefit of removing much of the inciting brolucizumab and proinflammatory intravitreal cytokines, patients who receive PPV do not demonstrate improved outcomes compared to their counterparts and are exposed to surgical risks [54,57]. However, limited data currently exist and definitive statements regarding the utility of this therapy cannot be made. When serum or vitreous cultures are obtained, they are negative by definition [57]. It is important to monitor retinal vasculitis patients closely to assess the severity of leakage and promptly identify occlusive events to allow for timely intervention. Repeat brolucizumab is contraindicated in an eye with active intraocular inflammation [65].

Final visual acuity (mean: 20/136 to 20/243, median: 20/80) is significantly worse than visual acuity at the time of the antecedent injection (mean: 20/52 to 20/53, median: 20/50) [54,57]. Eyes with vasculitis causing arterial occlusion proximal to the macula, optic nerve ischemia, or diffuse ischemia usually have visual acuity of 20/200 or worse at diagnosis and limited potential for vision improvement [57]. Milder cases generally have either non-occlusive retinitis or occlusion of a non-foveal vessel. While these cases generally have less severe visual acuity decline, it is unclear if prompt corticosteroid treatment leads to vision recovery or simply prevents progression.

### 4.4. Pathogenesis

The etiology of brolucizumab-associated retinal vasculitis remains unknown. The medication itself is unlikely to directly precipitate the vasculitis given that, as a single-chain antibody fragment, brolucizumab lacks an Fc region and thus cannot activate complement or participate in antibody-mediated cytotoxicity [66]. Although impurities related to drug manufacturing, storage, or delivery are known to cause SII [36,67,68,69], this is unlikely to account for the majority of BARV cases given that presentation is often delayed and most cases are sporadic rather clustered by drug lot or provider. One hypothesis proposes that brolucizumab’s smaller molecular weight relative to other anti-VEGF agents may allow for increased VEGF inhibition and deeper retinal penetration. This could diminish vascular perfusion in susceptible eyes, especially those with reduced retinal blood flow at baseline [57,70].

Alternatively, BARV may result from local anti-brolucizumab antibodies. This is supported by the European Medicines Agency’s Beovu public assessment report, which states that the presence of anti-brolucizumab serum antibodies are correlated with intraocular inflammation in clinical trials [71]. When anti-drug antibodies are present, systemic monoclonal antibody therapy for various oncologic diseases, including anti-VEGF agents, are known to induce a type III hypersensitivity reaction leading to vasculitis [72]. BARV may represent a similar type III hypersensitivity reaction wherein intravascular deposition of IgG/IgM complexes causes vasculitis and vascular occlusion. This would be similar to the pathogenesis of post-operative hemorrhagic occlusive retinal vasculitis (HORV), which is seen in some patients who receive intraocular vancomycin and have been exposed to vancomycin previously [73]. This comparison is strengthened by BARV and HORV’s similarities of delayed onset, intraocular inflammation, and retinal ischemia. However, there are also key differences, including a comparative lack of intraretinal hemorrhage at initial presentation in the former. This difference may be due to arterial occlusion, the anti-VEGF effect of brolucizumab, or that the two conditions are simply unrelated. Additional factors, such as HLA subtype, immune status, causative comorbidities, or prior exposure to compounds structurally similar to brolucizumab, may also factor into BARV pathogenesis [62].

### 4.5. Limitations and Guidelines

Current data are limited by small sample size, retrospective methodology, a lack of long-term follow-up, and variations in time to diagnosis, treatment regimen, and follow-up duration. Given the novelty of this condition and the limitations of existent data, further research is needed to better develop evidence-based treatment guidelines. As such, retina specialists are encouraged to report cases in as much detail as possible to the relevant clinical bodies. The MERLIN trial (ClinicalTrials.gov identifier: NCT03710564; https://clinicaltrials.gov/ct2/show/NCT03710564, accessed on 2 January 2021) is an ongoing prospective study that includes a more “real-world” sample of patients compared to HARRIER and HAWK, including many eyes that had previously received alternative anti-VEGF agents. As results of this trial become available, they may better elucidate the factors that predispose patients to developing BARV. Until such evidence is forthcoming, expert opinion has offered guidelines on managing these patients, including educating brolucizumab patients on urgent follow-up for relevant symptoms, evaluation for vasculitis or vascular occlusion in patients with post-injection intraocular inflammation, consideration of imaging modalities to aid in diagnosis, and aggressively treating affected patients with corticosteroids, and suspension of brolucizumab therapy while BARV is active [62].

## 5. Infectious Endophthalmitis

### 5.1. Epidemiology and Presentation

Infectious endophthalmitis remains one of the most devastating complications associated with anti-VEGF therapy. The reported incidence post-injection varies from 0.008 to 0.092%, although reported rates in recent studies are lower than those of their predecessors [74,75,76,77,78,79,80]. Despite its low incidence, the high volume of anti-VEGF injections means that they account for a large proportion of infectious endophthalmitis cases. A seven-year review of 199 endophthalmitis cases by Sachdeva et al. found that 8.5% of cases at a tertiary referral academic center followed anti-VEGF injections [81], while Gupta et al. found anti-VEGF injections accounted for 11% of their cases [82]. Infectious endophthalmitis has been reported with all available anti-VEGF agents, and its frequency does not differ significantly by drug [77,83,84,85].

Eyelid abnormalities such as ectropion are a risk factor for endophthalmitis and should be taken into account when considering treatment options in affected patients [75]. The use of post-injection topical antibiotics may also increase the risk of endophthalmitis and the prevalence of antibiotic resistance [75]. Other factors have been studied and found not to significantly affect the risk of endophthalmitis; these include age, sex, indication for anti-VEGF therapy, number of prior intravitreal injections, hemisphere of injection, conjunctival displacement, or bladed lid speculum use [30,86].

Symptoms develop between one and six days after the inciting injection (mean: 2.5 day), and presentation occurs at day three or four on average (range: one to 15 days) [30,81,87]. Decreased vision is present in virtually every patient (94–100%), as is pain (94–100%) [30,81]. This differs from SII and BARV, which have pain in 17 to 46% of patients [31,32,34,54,57]. The severity of pain also helps distinguish between these diseases, since pain is typically severe in endophthalmitis and mild-to-moderate in SII and BARV [31,32,34,54,57].

Visual acuity at presentation can vary from 20/80 to hand motion, but is worse than 20/100 in more than 80% of patients [81]. Conjunctival injection (100%), AC cell with hypopyon (78–100%), and vitritis (100%) are present on exam in most patients. Again, this differs from SII and BARV, in which injection is uncommon and hypopyon is rare [30,81].

### 5.2. Prevention

Prevention is key to reducing the incidence of infectious endophthalmitis. An outbreak of post-bevacizumab endophthalmitis resulted from poor aseptic technique [88,89]. This highlighted the importance of aseptic preparation of anti-VEGF syringes. Application of povidone–iodine to the conjunctival fornices is universally recommendation and the only topical prophylactic agent shown to reduce infectious endophthalmitis in prospective studies [90]. Use of sterile gloves is recommended as part of universal precautions, whereas placement of a sterile drape is optional [75]. Use of a sterile lid speculum is recommended during the injection to prevent needle contact with the lids or lashes. Face masks are recommended and talking should be limited as much as possible [74,91,92]. Topical antibiotics are used by some and have been shown to reduce ocular surface bacteria, but they have not demonstrated a significant effect on the rate of infectious endophthalmitis [75]. Some evidence even suggests antibiotic drops lead to increased antibiotic resistance of ocular flora and higher rates of endophthalmitis [93,94,95].

### 5.3. Treatment and Outcomes

Vitreous should be sent in cases of suspected endophthalmitis. If a suitable vitreous sample cannot be acquired during “tap and inject” (needle-based vitreous sampling for microbiologic analysis and intravitreal injection of antibiotics), then an aqueous sample should be sent instead via AC tap. If the patient is treated with prompt primary PPV, then the vitreous sample can be acquired at that time. Culture positivity varies by study, from 30 to 75% [30,81]. Coagulase-negative staphylococcal species are the most common isolates, found in 43 to 75% of culture-positive cases [30,79,81,83]. Streptococcus-associated endophthalmitis is the second most commonly identified pathogen and is associated with worse visual outcomes [30,79,83,96]. Unlike post-operative endophthalmitis, *Propionibacterium acnes* is exceedingly rare in post-injection cases [81]. Atypical pathogens should be considered in cases of dense, persistent vitritis in spite of antibiotic therapy and requiring PPV [81].

Retina specialists often use visual acuity measurements at the time of presentation to guide between tap/inject versus primary vitrectomy; this is based on findings from the Endophthalmitis Vitrectomy Study (EVS) [97]. However, the EVS was published in 1995 and comprised exclusively of post-operative endophthalmitis cases following cataract surgery or secondary intraocular lens implantation. Thus, EVS may have limited applicability to today’s post-injection endophthalmitis patient given advances in surgical technique and differences in the microbiology of post-operative and post-injection endophthalmitis. For instance, streptococcal species are more commonly implicated in post-injection endophthalmitis than post-operative cases [74,96,98,99]. This is likely due to a higher rate of iatrogenic infection from oral flora in injection settings compared to the sterile conditions of an operating room. This theory is supported by similar findings when comparing infectious endophthalmitis post-PPV versus post-injection [98]. A seven-year review of endophthalmitis cases at a tertiary referral academic center found that visual acuity measures did not predict success with either tap/inject or PPV, and that all patients did well with initial tap/inject, although some cases required subsequent PPV [81]. Similarly, Chaudhary et al. also found no significant difference in post-injection endophthalmitis outcomes between primary tap/inject and primary PPV [100].

When identified promptly and treated aggressively, visual acuity can return to baseline in up to 78% of patients [30,94]. Most patients who return to baseline visual acuity usually do so within three months, although this can take up to six months in some [30]. However, 22% of cases lose two or more lines of visual acuity permanently. Visual acuity loss can be profound, especially in cases of delayed treatment or atypical organisms, and at times include declines from normal or near-normal acuity to hand motion (HM), light perception (LP), or non-light perception (NLP) vision. Culture positivity is not significantly associated with severity of visual acuity decline, the rate of visual recovery, or the final visual acuity outcome [30,81]. Baseline visual acuity is also not predictive of full visual recovery [81]. Cases of *Streptococcus* endophthalmitis tend to display more rapid onset and poorer outcomes [30,79,81,96]. *Propionibacterium acnes* positivity is also associated with poor outcomes in infectious endophthalmitis, but it is a significantly rarer pathogen in post-injection endophthalmitis than in post-operative endophthalmitis [81]. Poor visual outcomes can result from various sequelae of infection, including retinal detachment, infection-related retinal necrosis, apoptosis of ganglion cells, bipolar cells, or photoreceptors, and hypotony [30,101]. In unfortunate cases, phthisis bulbi can develop, or chronic pain in a non-seeing eye may necessitate removal of the eye.

## 6. Discerning between SII, BARV, and Infectious Endophthalmitis

SII, BARV, and infectious endophthalmitis are difficult to discern from one another. They are all inflammatory complications that arise following intravitreal administration of anti-VEGF agents and present with a similar constellation of symptoms and exam findings.

It is unclear if nuanced differences in the presentation of SII and infectious endophthalmitis are useful in differentiating the two conditions. Mezad-Koursch et al. suggested that an increased severity of clinical findings, the presence of pain, and a longer time to presentation are helpful in discerning infectious endophthalmitis from SII [87]. Another study observed that none of their 56 SII subjects demonstrated conjunctival injection, and severe pain and hypopyon were each only seen in one patient [34]. This differs from infectious endophthalmitis—in which severe pain, injection, and hypopyon are each hallmark features—and suggests that SII could be cautiously considered in cases with rapid post-injection presentation and the absence of pain, injection, and hypopyon. Guidelines have been published to distinguish between the two entities, largely based on similar guidelines for distinguishing infectious endophthalmitis from SII following intravitreal triamcinolone acetate administration [102].

However, other studies have not found these or other features to be reliable in differentiating between SII and endophthalmitis [30,103]. Given the lack of definitive methods for differentiating the two conditions and the necessity to treat infectious endophthalmitis promptly and aggressively, it is reasonable to default towards approaching each case as infectious unless a diagnosis of SII is deemed highly likely. However, in cases where inflammation is relatively light, it is also reasonable to treat with topical corticosteroids and monitor closely.

Similarly, BARV and infectious endophthalmitis must both be considered in patients who demonstrate intraocular inflammation with vasculitis or retinal vascular occlusion after intravitreal injection of brolucizumab. Clinical features that are common in infectious endophthalmitis but rare in BARV include severe pain, conjunctival injection, and hypopyon. Endophthalmitis also has no sex predilection, whereas BARV has a strong female predominance (>80%) [54,57]. These features may help guide diagnosis in post-brolucizumab patients with evidence of vasculitis or vascular occlusion. However, these factors should be used cautiously since similar measures to distinguish between SII and endophthalmitis have shown mixed results. It is essential that the full clinical picture along with one’s clinical experience and judgment be used when trying to establish the diagnosis and treatment plan.

Patients who have intraocular inflammation after intravitreal brolucizumab injection but with no signs of vasculitis should still be assessed for all three diagnoses. So long as no evidence of vasculitis or vascular occlusion is present on exam or FA, BARV cannot be diagnosed. However, some BARV patients only develop signs of vasculitis or vascular occlusion later in their disease course. Thus, a patient in this situation who is deemed to not have endophthalmitis may be considered to likely have SII, but they should be monitored closely with serial dilated exams and FA with peripheral sweeps to assess for delayed onset of vasculitis or vascular occlusion.

## 7. Conclusions

SII, BARV, and infectious endophthalmitis are uncommon adverse events associated with intravitreal anti-VEGF administration. SII and BARV are generally characterized by blurring of vision, floaters, and minimal-to-no pain. However, BARV is specific to brolucizumab, whereas SII can be associated with any anti-VEGF agent. BARV—a diagnosis that only became apparent after post-market surveillance and post hoc analysis of the Phase 3 HAWK and HARRIER trials—requires evidence of retinal vasculitis or vascular occlusion to be diagnosed, whereas these findings are not seen in SII. Topical corticosteroids are the most common treatment regimen for SII, and outcomes are generally good. Corticosteroid treatment is typically more aggressive in BARV and the visual prognosis is more guarded, especially if vasculitis causes arterial occlusion proximal to the macula, optic nerve ischemia, or diffuse ischemia. The presentations of these conditions are similar to that of infectious endophthalmitis. Given the potentially devastating outcomes in infectious endophthalmitis and the need to treat promptly and aggressively, it must be thoroughly considered in any patient with post-injection intraocular inflammation. The presentation of infectious endophthalmitis differs in that it usually features severe pain, conjunctival injection, and hypopyon. Some guidelines have been proposed based on these differences to help discern between SII or BARV and infectious endophthalmitis, but there is evidence that these guidelines are not definitive. Retina specialists should still rely on clinical judgement and expertise when treating SII or BARV, and there should be a low threshold to treat a case as possibly infectious if there is diagnostic uncertainty.

## Data Availability

Not applicable.
